# Unusual type of diabetes: fibrocalculous pancreatic diabetes

**DOI:** 10.11604/pamj.2021.38.116.21663

**Published:** 2021-02-03

**Authors:** Natnicha Houngngam, Thiti Snabboon

**Affiliations:** 1 Excellence Center in Diabetes, Hormone and Metabolism, King Chulalongkorn Memorial Hospital, Thai Red Cross Society, Bangkok, Thailand,; 2 Department of Medicine, Faculty of Medicine, Chulalongkorn University, Bangkok, Thailand

**Keywords:** Fibrocalculous pancreatic diabetes, tropical chronic pancreatitis, ketosis-resistant diabetes

## Image in medicine

A 34-year-old Thai man complained about recurrent upper abdominal pain, polyuria and a 5kg weight loss in 6 months. He had no history of steatorrhea, jaundice, gallstones, alcohol intake, or cassava consumption. Physical examination was unremarkable except for his body mass index of 17.6 kg/m^2^. The plasma glucose level was 672 mg/dL with normal serum amylase and lipase. Laboratory investigation showed serum osmolality of 287 mOsm/kg, bicarbonate 22 mEq/L and negative for serum ketone. Fibrocalculous pancreatic diabetes (FCPD) was diagnosed from a plain abdominal X-ray study showing multiple large calcifications over a pancreatic area (A). Computerized tomography of the abdomen revealed a large tubular calcification in dilated pancreatic duct during entering the duodenum (B). Scattered calcifications along the pancreatic duct were also noted (C). A genetic study revealed a SPINK1 N34S heterozygous mutation. His hyperglycemia responded well to insulin therapy. FCPD, a late stage of tropical chronic pancreatitis (TCP), is classified as a secondary cause of diabetes resulting from pancreatic dysfunction. The clinical picture consists of a triad of pancreatic calcification, abdominal pain, and diabetes. Its distinctive features are young age at onset, lean or underweight, ketosis-resistant diabetes, presence of large intraductal pancreatic calculi, and reported mainly in tropical developing countries. An etiology was previously thought to relate to malnutrition; however, a strong association with SPINK1 mutation is noted. The major morbidities are recurrent abdominal pain, steatorrhea, and malnutrition, while the majority of death is associated with diabetic nephropathy and pancreatic cancer. Treatment includes pancreatic enzyme supplementation, insulin therapy, and surgical drainage.

**Figure 1:**
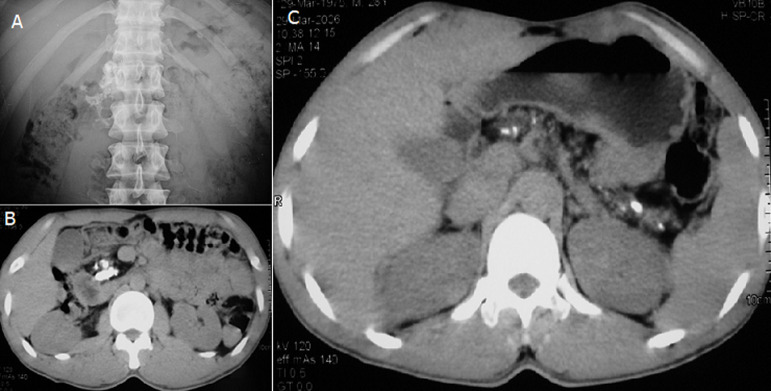
plain abdominal X-ray showed multiple large calcifications over pancreatic area (A), computerized tomography revealed a large tubular calcification in dilated pancreatic duct (B) and small scattered calcifications along pancreatic shadow (C)

